# Effects of Physiological Load on Kinematic Variables Related to Tennis Serve Performance

**DOI:** 10.3390/sports13070197

**Published:** 2025-06-22

**Authors:** Zlatan Bilić, Mateja Očić, Vedran Dukarić, Lidija Petrinović, Petar Barbaros

**Affiliations:** Faculty of Kinesiology, University of Zagreb, 10000 Zagreb, Croatia; mateja.ocic@kif.unizg.hr (M.O.); vedran.dukaric@kif.unizg.hr (V.D.); lidija.petrinovic@kif.unizg.hr (L.P.); petar.barbaros@kif.unizg.hr (P.B.)

**Keywords:** physiological fatigue, inertial measurement units, serve speed, serve accuracy, kinetic chain

## Abstract

This study aimed to explore the effects of fatigue on the kinematic parameters and performance of the tennis serve, specifically speed and accuracy. Xsens inertial measurement units (IMUs) were used to obtain the kinematic parameters of the tennis serve. Seven professional tennis players participated in the study, performing multiple sets of serves along with tennis-specific fatigue-inducing drills. Key kinematic variables such as jump height, pelvis velocity, wrist height, wrist velocity, upper arm velocity, forearm velocity, and shoulder velocity were measured alongside serve speed and accuracy. Results indicated significant fatigue-induced alterations in several kinematic variables, notably jump height and wrist velocity, which affected the serve performance. Importantly, serve speed and accuracy declined in the final sets, correlating with increased heart rate and fatigue. These findings provide insights into the biomechanical effects of fatigue on the serve, with implications for training and performance optimization.

## 1. Introduction

The serve is considered one of the most important strokes in modern tennis [1,2,3,4,5,6], and the quality of its execution is a key component of successful play. As the stroke that initiates every point and accounts for approximately 60% of all strokes during a match, the serve is arguably the most dominant and decisive action in the game [1]. From a biomechanical perspective, the tennis serve is a complex movement involving a sequence of coordinated actions throughout the kinetic chain, from the feet, through the legs and trunk, to the upper limbs, requiring precise technical execution [7]. Effective serving relies on the synchronized activation of selected muscle groups, segmental rotations, and the coordinated motion of the lower and upper body [8,9]. Coordination of the kinetic chain is essential not only for optimizing energy transfer into the ball but also for maximizing both speed and precision during the serve. Several studies have analyzed the kinematic phases of the tennis serve, often focusing on specific joint angles at key moments such as maximal knee flexion, shoulder external rotation, or ball impact [8,10,11,12]. As the starting point of the kinetic chain, the role of the lower limbs is particularly emphasized, as leg drive significantly influences trunk and arm kinematics [13,14]. Given the complexity of the serve, players must develop a high level of motor precision to maintain performance and avoid technical breakdowns, especially under conditions of physical fatigue [7]. Serve success is highly dependent on both speed and accuracy. A fast serve places temporal pressure on the opponent, while precision determines point effectiveness [15]. Among senior-level players, serve speed is widely considered a critical performance factor [4,5,16,17], yet even minor deviations in ball placement, often within just a few centimeters, can distinguish a successful from an unsuccessful attempt [18]. The ability to consistently achieve high speeds with precision serves as a distinguishing factor among elite players.

One of the primary factors influencing the decrease in motor performance in sport is fatigue, which results from physiological stress on the body’s systems [19]. To evaluate cardiac activity based on physiological responses induced by fatigue, this study employed one of the most used methods, heart rate monitoring [20]. Previously, studies examined physiological responses to the demands of intensive tennis training by analyzing heart rate as a key variable [21,22,23]. Furthermore, research has investigated the effects of fatigue on serve speed [17,24] and accuracy [25,26] using different testing protocols, consistently demonstrating a negative impact on serve performance under fatigued conditions [27,28,29]. Fatigue induced variability in motor control and may lead to a decrease in the biomechanical efficiency of the serve, thus affecting both speed and accuracy [30].

To investigate these effects, researchers have commonly employed 2D and 3D video-based motion analysis systems, both marker-based and markerless, to capture detailed movement data [31,32,33]. While 3D motion capture is considered the gold standard for kinematic analysis [34,35,36], its application in real-world tennis settings remains limited due to environmental constraints and the complexity of movements [37,38]. As a practical and non-invasive alternative, inertial measurement units (IMUs), such as those provided by Xsens, allow for reliable tracking of joint positions, accelerations, and angular velocities during dynamic tasks in real time [39,40,41,42,43]. Their wearable design and mobility make them ideal for field-based assessments, enabling researchers and coaches to analyze performance outside of laboratory conditions. However, despite their proven utility, few studies have focused on the use of such technology to evaluate the tennis serve under physiological load, especially concerning accuracy and velocity. Although previous research has extensively examined the biomechanics of the tennis serve, there is a lack of studies analyzing how physiological load (i.e., fatigue) influences both kinematic indicators and performance outcomes such as serve speed and accuracy. Furthermore, the application of IMU-based systems such as Xsens in capturing movement adaptations under realistic, sport-specific conditions remains largely unexplored. Therefore, this study aims to investigate the kinematic indicators of serve performance under conditions of physiological stress, with a specific focus on accuracy and speed, using advanced motion capture technology.

## 2. Materials and Methods

### 2.1. Participants

The sample consisted of seven professional tennis players (age: 22.29 ± 1.98 years; body height: 182.71 ± 4.86 cm; body mass: 75.86 ± 4.06 kg) who regularly compete in professional tennis tournaments at the ITF and ATP levels. Participants were recruited using a non-probabilistic convenience sampling method based on their availability and existing collaboration with the research institution. This approach was considered appropriate given the performance requirements and the high physical demands of the testing protocol. All participants met the inclusion criteria of being healthy, injury-free for at least six months prior to the study, and regularly training 4–5 times per week. The study was conducted in accordance with the Declaration of Helsinki and approved by the Ethics Committee of the Faculty of Kinesiology, University of Zagreb (Approval No. 50/2025). To determine the appropriate sample size, a G*Power analysis was conducted (version 3.1.9.4; Heinrich Heine University, Düsseldorf, Germany), indicating that a sample of seven participants was sufficient to achieve an effect size of f = 0.85, with an alpha level of 0.05 and a statistical power of 0.90.

### 2.2. Instruments

**Figure 1:**
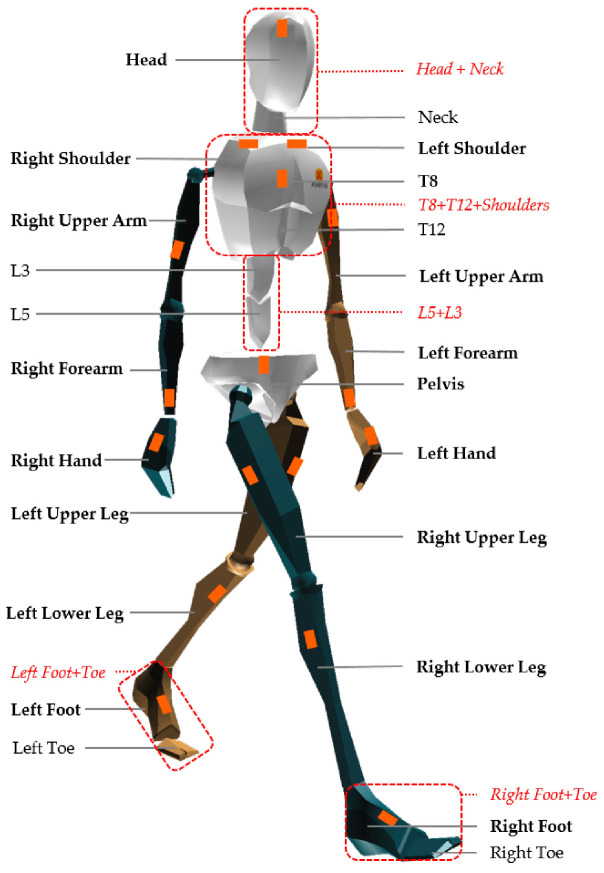
Sensor positioning for motion capture [47].

To measure body height and body weight, a portable stadiometer SECA 213 (Seca GmbH & Co. KG, Hamburg, Germany) and a body composition scale TANITA BC-545N (Tanita Corporation, Tokyo, Japan) were used. To analyze the kinematic parameters of the serve, the MNV BIOMECH Awinda inertial measurement system (Xsens Technologies B.V., Enschede, The Netherlands) was utilized. The system calibration procedure (i.e., sensor-to-body alignment and body dimension determination) was performed according to the manufacturer’s instructions [43]. Each player wore a full-body suit equipped with 17 wireless inertial sensors (Figure 1), sampling at 60 Hz, allowing for detailed three-dimensional motion capture. Calibration of the sensors was performed in an N-pose. Movement data were processed using the MVN Studio BIOMECH software (Xsens Technologies B.V., Enschede, The Netherlands). Previously [44,45,46], the reliability and validity of motion capture with Xsens were determined in specific conditions and functional activities.

Heart rate was monitored using the H10 heart rate sensor (Polar Electro, Kempele, Finland), a validated device for tracking heart rate variability [48,49].

Serve speed was measured using the Stalker Pro radar gun (Applied Concepts Inc., Richardson, TX, USA), validated for measuring ball velocities across various sports [50]. This method was conducted according to a previously established protocol [51]. Briefly, the radar was positioned 3 m behind the server, facing the center of the baseline, aligned with the estimated ball contact height (~2.2 m), and pointed straight down the centerline of the court.

### 2.3. Variables

The variables analyzed in the present study consisted of parameters for evaluating the first flat serve. During the vertical jump, the height of the jump (Jump_H) was measured from the loading position to the moment of ball–racket contact. The height of the wrist during the serve (Wrist_H) was recorded. Throughout the acceleration and follow-through phases of the serve, segmental velocities were assessed, including those of the pelvis (Pelvis_V), wrist (Wrist_V), upper arm (Upper_arm_V), forearm (Forearm_V), and shoulder (Shoulder_V)

Serve Accuracy (SA) was assessed using a 0–6 point scale, where 0 represented the lowest and 6 the highest accuracy. The total accuracy score was calculated after each set of five serves. Serve Speed (SP) was measured in kilometers per hour (km/h). The average serve speed was calculated for each set of ten serves.

The average heart rate (HR) during serving was calculated for each series.

### 2.4. Overview of Measurement Procedures

For the purpose of serve accuracy testing, target zones on the deuce side of the court, based on the Hewitt Tennis Achievement Test, were used, as applied in similar studies [52,53]. Each serve was awarded a specific number of points depending on the target zone hit (Figure 1). The name of each zone corresponded to its point value (1, 2, 3, 4, 5, or 6). If the serve resulted in a fault (i.e., net or out), zero points were assigned.

**Figure 2:**
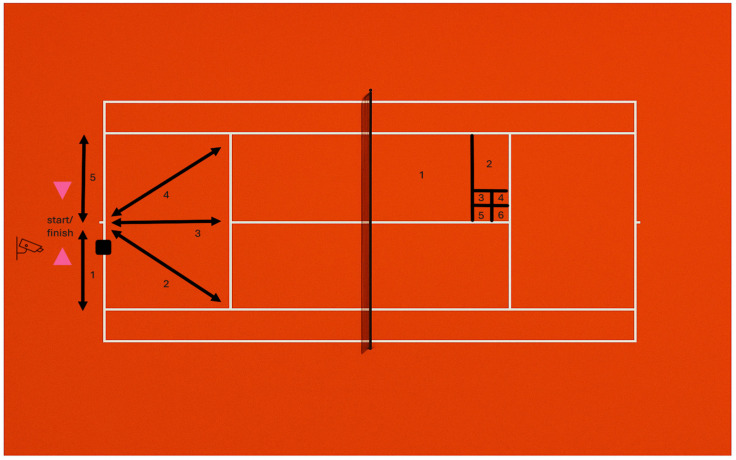
Server position (▪) and accuracy zones (1, 2, 3, 4, 5, 6).

The spider drill test was used to induce tennis-specific fatigue between serve series. The spider is a reliable test for assessing change in direction speed (CODS) in tennis players [54]. Participants started the test by breaking the timing gate beam and performing five sprints in a specific sequence (Figure 2), beginning to the right and continuing counterclockwise. Distances ranged from 4.11 to 5.49 m. After each sprint, players returned to the center point. The test concluded with a final 3-m sprint through the timing gates following a 90° right turn.

### 2.5. Experimental Protocol

Before the start of testing, all participants completed a standardized, tennis-specific warm-up lasting approximately 15 min. The warm-up consisted of low-intensity aerobic running (5 repetitions of 20-m lengths), followed by dynamic stretching exercises aimed at activating major muscle groups and enhancing movement readiness. To prepare for the serving component of the testing protocol, each participant performed 30 first serves at a self-selected pace and intensity.

Upon completion of the warm-up, participants were fitted with the H10 heart rate monitor and the full-body kinematic sensor system. The testing protocol followed a repeated sequence of serve execution and change in direction assessment. Specifically, each participant completed six consecutive blocks, each consisting of 10 serves, followed immediately by the spider drill. The structure was as follows: 10 serves → spider drill → 10 serves → spider drill → 10 serves → spider drill → 10 serves → spider drill → 10 serves → spider drill → 10 serves.

Participants were instructed to perform the entire testing protocol at maximal effort. Each serve within a series was executed with minimal rest between attempts to simulate match-like intensity and induce physiological fatigue. Following the completion of each spider drill, participants immediately returned to the service line without rest to begin the next set of serves, maintaining a continuous and demanding workload throughout the testing session. According to the instructions, the players served the first flat serve, as they would in matches. Training intensity was ensured by requiring maximal effort during each serve and sprint, with minimal recovery to simulate match conditions. Fatigue was monitored individually using heart rate (targeting > 85% HRmax) and performance decline in serve speed and accuracy.

This testing protocol was designed to assess variations in serve performance and changes in direction speed under increasing physiological load while simultaneously capturing physiological (heart rate) and kinematic data in real time.

### 2.6. Statistical Analysis

Descriptive statistics (mean, standard deviation) were calculated for each kinematic variable and performance parameter (serve speed, accuracy, heart rate). The normality of the distribution was tested with the Shapiro–Wilk test. To explore the effects of fatigue on the observed variables, a MANOVA for repeated measures was performed. The overall multivariate effect was determined. Post-hoc comparisons were carried out using the Tukey test to determine which interactions were statistically significant. Statistical analysis was performed with the use of Statistica 14.0.1.25 (TIBCO software, Inc., Palo Alto, CA, USA). The level of statistical significance was set at *p* < 0.05.

## 3. Results

Table 1 presents the basic descriptive parameters of the observed variables. It is notable that Jump_H values progressively decreased with repetitions (from 20.03 cm to 15.38 cm), while variability, as indicated by standard deviation, progressively increased. The highest velocity among the observed segments was recorded in Wrist_V, with a peak value of 13.24 m/s in the second set of serves. Pelvis_V ranged from 1.06 m/s to 2.59 m/s. The maximum measured wrist height was 229.54 cm in the first condition, while the maximum recorded serve speed was 203.00 km/h.

MANOVA for repeated measures was used to determine differences between serving variables after the fatigue protocol. There is a significant difference observed in the overall effects of the variables across different conditions (F = 13.86; *p* = 0.00).

The post hoc test (Table 2) presents differences in serve performance under the influence of fatigue. In Jump_H, the final set of serves showed significant differences compared to all other conditions, indicating that this variable is highly sensitive to increased fatigue. Pelvis_V exhibited a pattern of significant variability across multiple sets. Wrist velocity (Wrist_V) showed the most prominent changes in the third set, while wrist height (Wrist_H) differed significantly between the first two and the final condition. The variables Upper_arm_V and Forearm_V demonstrated only minor differences across interactions. Shoulder velocity (Shoulder_V) was found to be most affected by fatigue, with the final condition differing significantly from all others except the first. Furthermore, serve speed (Serve_V) showed a statistically significant difference in interaction 6.

Serve accuracy and mean heart rate during serving are presented in Table 3. The highest accuracy was recorded in the second series (21.00), while the lowest was observed in the fourth series (14.71). The lowest average heart rate (HR) was recorded in the first interaction (147.43 bpm), while the highest was observed in the final interaction (164.71 bpm).

## 4. Discussion

The primary aim of this study was to analyze how physiological fatigue affects the kinematic indicators and performance parameters of the tennis serve, with a focus on changes in serve speed and accuracy. The results obtained indicate significant changes in several kinematic variables that form the kinetic chain of the serve, with the most noticeable changes occurring at higher levels of fatigue. In general, our kinematic data are comparable to previous findings [55,56,57]. The most pronounced effect of fatigue was observed in the progressive decrease in jump height (Jump_H) during the serve series. The reduction in vertical explosiveness suggests a decreased ability of the lower extremities to generate the required force for the initial swing. This is particularly important, as the lower extremities are considered the foundation of the kinetic chain, which extends through the torso, shoulders, and arm. Knee flexion and hip engagement play a crucial role in the vertical component of the serve, and their reduction directly decreases the potential for energy transfer to the upper segments [40]. Based on a higher jump height, players create the potential for a higher contact point with the ball. This biomechanical advantage helps them achieve greater serve speed: with a higher contact point, the player generates a greater peripheral racket speed in the moment of ball impact compared to a lower contact point, even when the angular velocity of the upper extremity segments is the same. This also results in a greater torque at the hand-racket joint [58]. The stability of the player’s vertical movement towards contact with the ball is consistent with previous research that emphasized its importance for serve coordination [34]. Pelvis velocity (Pelvis_V), which represents the central point of force transfer between the lower and upper body, also showed variability due to fatigue. One of the primary energy generators in the serve performance is the timely and powerful rotation of the pelvis [8]. If this rotation is slowed down or insufficiently synchronized, energy transfer through the kinetic chain is compromised, reducing movement efficiency and, consequently, serve speed. Changes in wrist velocity (Wrist_V) are particularly noticeable during the middle stages of the protocol, suggesting an early tendency of the distal segments to fatigue. The wrist and elbow flexion is strongly correlated with racket speed in the moment of ball contact, and fatigue in these areas can lead to reduced control, later ball contact, and impaired shot accuracy [40]. Furthermore, changes in wrist height (Wrist_H) further indicate compensations made by athletes during fatigue, such as improper positioning of the hitting arm. In contrast to the notable changes in the distal segments, the velocities of the upper arm (Upper_arm_V) and forearm (Forearm_V) remained relatively stable. Although the differences were small, even minimal changes in the arm segments can have a cumulative effect on the final racket speed and, consequently, on serve speed [8]. A possible explanation for the stability of these variables lies in the players’ ability to compensate for the decline in performance of the distal joints using the proximal segments more stably, as well as the technical quality of the serve execution, given that these are elite senior players. Shoulder velocity (Shoulder_V) showed relatively inconsistent results across all serve series, with the lowest average values progressively occurring during the 3rd, 4th, and 5th series. This may be a result of fatigue accumulated from the previous serve series, particularly through the initial segments of the kinetic chain (lower extremities and torso), which ultimately places additional strain on the shoulder as the final point of force before ball contact.

Serve velocity (Serve_V) shows a significant decrease in the final serve series, with a continuous decline in the average serve speed from the 4th to the 6th series. This confirms the relationship between disrupted segmental coordination and the final outcome of the shot caused by fatigue. This result is consistent with previous research that highlights how fatigue reduces the efficiency of the kinetic chain, leading to asynchrony, decreased force transfer, and weaker performance outcomes in terms of speed [40]. In addition to biomechanical factors, it is possible that the decrease in serve speed is also influenced by a reduced perception of control over the movement and an increased need to maintain stability during the serve under fatigue.

Based on the presented results, it can be observed that the average heart rate (HR) progressively increased from the first series (147.43 bpm) to the final series (164.71 bpm) as the number of series increased. This suggests that a progressive loading effect was achieved, which was the intended goal of the experimental protocol. A similar trend was reported in previous research, where a progressive increase in heart rate was observed during tennis training sessions as the number of strokes increased [59].

In the serve accuracy (SA) parameter, it can be observed that the results vary across the serve series. Overall, players’ performance was poor, with their best result in the 2nd series (21 points) and their worst in the 4th series (14.71), out of a total of 60 possible points. When comparing these results to previous research [29] with a similar testing protocol, contrasting results were found, where accuracy significantly decreased after fatigue induced by a specific high-intensity test. A possible explanation for this outcome may lie in the sample of participants, where despite accumulated fatigue, they maintained a level of accuracy without the progressive decline seen in previous research, where professional players at the highest competition level were able to maintain their serve accuracy despite accumulated fatigue [60].

The significance of this study is twofold: on the one hand, it provides a deeper understanding of the kinematic parameters of the serve under the influence of fatigue, and on the other, it offers practical guidelines for the training process. However, it is important to highlight certain limitations of this study. A methodological limitation of this study is the use of a non-probabilistic convenience sample. While all participants were professional-level tennis players, this sampling method may limit the generalizability of the findings and may affect the representativeness of the sample. Future studies with larger and more diverse samples recruited via probabilistic methods would enhance the external validity of the results. Despite this, the differences observed were statistically significant. Future research should include a larger number of participants, which would allow for a comparison between higher-ranked and lower-ranked tennis players. Furthermore, future research following a similar protocol could measure serve speed, evaluate the technical performance of the serve by tennis experts, and conduct both control and experimental measurements on the same participants to obtain more robust results for the observed serve-related parameters.

## 5. Conclusions

This study highlights the significant impact of physiological fatigue on the kinematic efficiency and performance of the tennis serve. Fatigue led to reductions in key performance indicators such as serve speed and accuracy, particularly affecting the lower body’s contribution to the kinetic chain, which in turn compromised the overall effectiveness of the serve. These findings are valuable for tailoring training programs that account for fatigue management, ensuring that athletes can maintain performance under competitive conditions.

## Figures and Tables

**Table 1:** Descriptive data of the observed variables.

**Var**	**Jump_H**		**Pelvis_V**		**Wrist_H**		**Wrist_V**	
**Rep**	**Mean**	**St.dev**	**Mean**	**St.dev**	**Mean**	**St.dev**	**Mean**	**St.dev**
1	20.03	5.56	1.89	0.27	213.22	11.16	8.91	1.51
2	19.81	5.64	1.94	0.26	211.62	13.09	8.63	2.06
3	18.83	6.36	1.96	0.28	210.41	12.99	8.00	1.98
4	18.36	6.05	1.95	0.24	210.93	12.46	8.41	1.97
5	17.26	6.37	1.88	0.30	211.47	10.65	8.43	2.16
6	15.38	7.54	1.86	0.29	209.71	12.52	9.13	2.10
**Var**	**Upper_arm_V**		**Forearm_V**		**Shoul_V**		**Serve_V**	
**Rep**	**Mean**	**St.dev**	**Mean**	**St.dev**	**Mean**	**St.dev**	**Mean**	**St.dev**
1	3.32	0.45	7.30	0.98	2.58	0.65	178.44	8.46
2	3.26	0.51	7.00	1.13	2.39	0.71	179.63	10.63
3	3.27	0.77	6.70	1.81	2.35	0.81	180.87	9.47
4	3.06	0.86	6.38	2.33	2.31	0.88	179.24	10.66
5	3.33	0.56	6.59	1.45	2.38	0.69	176.37	11.49
6	3.37	0.63	6.84	1.50	2.65	0.83	175.16	12.22

Legend: Rep—number of serve series; Mean—average values; Min—minimum value; Max—maximum value; St.dev.—standard deviation; Jump_H—height of the jump; Pelvis_V—velocity of pelvis; Wrist_H—height of the wrist during serve; Wrist_V—velocity of the wrist; Upper_arm_V—velocity of the upper arm; Forearm_V—velocity of the forearm; Shoul_V—velocity of the shoulder; Serve_V—velocity of ball.

**Table 2:** Tukey post hoc test for each variable.

	**Jump_H**							**Pelvis_V**					
**Interaction**	**{1}**	**{2}**	**{3}**	**{4}**	**{5}**	**{6}**	**Interaction**	**{1}**	**{2}**	**{3}**	**{4}**	**{5}**	**{6}**
1		1.00	0.25	0.03 *	0.00 *	0.00 *	1		0.05	0.00 *	0.02 *	1.00	0.62
2	1.00		0.49	0.09	0.00 *	0.00 *	2	0.05		0.97	1.00	0.03 *	0.00 *
3	0.25	0.49		0.96	0.05	0.00 *	3	0.00 *	0.97		1.00	0.00 *	0.00 *
4	0.03 *	0.09	0.96		0.34	0.00 *	4	0.02 *	1.00	1.00		0.01 *	0.00 *
5	0.00 *	0.00 *	0.05	0.34		0.01 *	5	1.00	0.03 *	0.00 *	0.01 *		0.77
6	0.00 *	0.00 *	0.00 *	0.00 *	0.01 *		6	0.62	0.00 *	0.00 *	0.00 *	0.77	
	**Wrist_H**							**Wrist_V**					
**Interaction**	**{1}**	**{2}**	**{3}**	**{4}**	**{5}**	**{6}**	**Interaction**	**{1}**	**{2}**	**{3}**	**{4}**	**{5}**	**{6}**
1		0.10	0.00 *	0.00 *	0.05	0.00 *	1		0.91	0.02 *	0.48	0.53	0.98
2	0.10		0.37	0.88	1.00	0.03 *	2	0.91		0.24	0.97	0.98	0.49
3	0.00 *	0.37		0.96	0.52	0.87	3	0.02 *	0.24		0.71	0.67	0.00 *
4	0.00 *	0.88	0.96		0.95	0.36	4	0.48	0.97	0.71		1.00	0.12
5	0.05	1.00	0.52	0.95		0.05	5	0.53	0.98	0.67	1.00		0.14
6	0.00 *	0.03 *	0.87	0.36	0.05		6	0.98	0.49	0.00 *	0.12	0.14	
	**Upper_arm_V**							**Forearm_V**					
**Interaction**	**{1}**	**{2}**	**{3}**	**{4}**	**{5}**	**{6}**	**Interaction**	**{1}**	**{2}**	**{3}**	**{4}**	**{5}**	**{6}**
1		0.99	1.00	0.12	1.00	1.00	1		0.73	0.06	0.00 *	0.01 *	0.25
2	0.99		1.00	0.38	0.99	0.91	2	0.73		0.73	0.04 *	0.38	0.97
3	1.00	1.00		0.33	0.99	0.94	3	0.06	0.73		0.64	0.99	0.99
4	0.12	0.38	0.33		0.11	0.04 *	4	0.00 *	0.04 *	0.64		0.92	0.26
5	1.00	0.99	0.99	0.11		1.00	5	0.01 *	0.38	0.99	0.92		0.86
6	1.00	0.91	0.94	0.04 *	1.00		6	0.25	0.97	0.99	0.26	0.86	
	**Shoul_V**							**Serve_V**					
**Interaction**	**{1}**	**{2}**	**{3}**	**{4}**	**{5}**	**{6}**	**Interaction**	**{1}**	**{2}**	**{3}**	**{4}**	**{5}**	**{6}**
1		0.21	0.05	0.01 *	0.17	0.96	1		0.76	0.07	0.94	0.18	0.00 *
2	0.21		0.99	0.91	1.00	0.03 *	2	0.76		0.72	1.00	0.00 *	0.00 *
3	0.05	0.99		1.00	1.00	0.00 *	3	0.07	0.72		0.44	0.00 *	0.00 *
4	0.01 *	0.91	1.00		0.94	0.00 *	4	0.94	1.00	0.44		0.01 *	0.00 *
5	0.17	1.00	1.00	0.94		0.02 *	5	0.18	0.00 *	0.00 *	0.01 *		0.74
6	0.96	0.03 *	0.00 *	0.00 *	0.02 *		6	0.00 *	0.00 *	0.00 *	0.00 *	0.74	

Legend: Jump_H—height of the jump; Pelvis_V—velocity of pelvis; Wrist_H—height of the wrist during serve; Wrist_V—velocity of the wrist; Upper_arm_V—velocity of the upper arm; Forearm_V—velocity of the forearm; Shoul_V—velocity of the shoulder; Serve_V—velocity of ball; * marked values are significant when *p* < 0.05.

**Table 3:** Results of serve accuracy and heart rate during serve series.

Series	SA	HR
1	18.43	147.43 bpm
2	21.00	156.00 bpm
3	20.43	161.00 bpm
4	14.71	164.14 bpm
5	19.43	164.29 bpm
6	15.29	164.71 bpm

Legend: Series—number of series; SA—serve accuracy; HR—average heart rate.

## Data Availability

The data presented in this study are available on request from the corresponding author. The data are not publicly available due to privacy concerns.
